# Spotlight influenza: The 2019/20 influenza season and the impact of COVID-19 on influenza surveillance in the WHO European Region

**DOI:** 10.2807/1560-7917.ES.2021.26.40.2100077

**Published:** 2021-10-07

**Authors:** Cornelia Adlhoch, Miriam Sneiderman, Oksana Martinuka, Angeliki Melidou, Nick Bundle, James Fielding, Sonja J Olsen, Pasi Penttinen, Lucia Pastore, Richard Pebody, the European Influenza Surveillance Network, Members of the European Influenza Surveillance Network, Members of the European Influenza Surveillance Network, Artan Simaku, Iris Hatibi, Monika Redlberger-Fritz, Veronika Vysotskaya, Natallia Shmialiova, Nathalie Bossuyt, Isabelle Thomas, Nina Rodić-Vukmir, Amela Dedeić-Ljubović, Neli Korsun, Antoaneta Minkova, Goranka Petrović, Irena Tabain, Martina Havlickova, Helena Jirincova, Jan Kyncl, Lasse Skafte Vestergaard, Ramona Trebbien, Olga Sadikova, Natalja Kuznetsova, Niina Ikonen, Outi Lyytikäinen, Shirley Masse, Vincent Enouf, Martine Valette, Irakli Karseladze, Mari Gavashelidze, Silke Buda, Ralf Dürrwald, Maria Exindari, Joan O'Donnell, Linda Dunford, Zalman Kaufman, Flavia Riccardo, Antonino Bella, Smagulova Meiramgul, Usserbayev Aidar, Otorbaeva Dinagul Satarovna, Ashyralieva Damira Omurzakovna, Raina Nikiforova, Natalija Zamjatina, Svajūnė Muralytė, Asta Skrickienė, Jackie Maistre Melillo, Tanya Melillo, Marit M.A. de Lange, Mariëtte Hooiveld, Gordana Kuzmanovska, Dragan Kochinski, Maja Kuzmanovska, Trine Hessevik Paulsen, Olav Hungnes, Ana Paula Rodrigues, Raquel Guiomar, Elena Burtseva, Kirill Stolyarov, Elizaveta Smorodintseva, Dragana Dimitrijevic, Maja Sočan, Katarina Prosenc, Jesús Oliva, Concepción Delgado-Sanz,, Amparo Larrauri, Mia Brytting, AnnaSara Carnahan, Ana Rita Gonçalves, Emine Avci, Ayse Basak Altas, Alla Mironenko,, Oksana Artemchuk, Iryna Demchyshyna, Mark O’Doherty, David Irwin

**Affiliations:** 1 European Centre for Disease Prevention and Control (ECDC), Stockholm, Sweden; 2 World Health Organization (WHO) Regional Office for Europe, Copenhagen, Denmark; 3 The members of the European Influenza Surveillance Network are listed under Investigators

**Keywords:** Europe, influenza, sentinel surveillance, epidemiology, COVID-19, 2019/20 season

## Abstract

**Background:**

Annual seasonal influenza activity in the northern hemisphere causes a high burden of disease during the winter months, peaking in the first weeks of the year.

**Aim:**

We describe the 2019/20 influenza season and the impact of the COVID-19 pandemic on sentinel surveillance in the World Health Organization (WHO) European Region.

**Methods:**

We analysed weekly epidemiological and virological influenza data from sentinel primary care and hospital sources reported by countries, territories and areas (hereafter countries) in the European Region.

**Results:**

We observed co-circulation of influenza B/Victoria-lineage, A(H1)pdm09 and A(H3) viruses during the 2019/20 season, with different dominance patterns observed across the Region. A higher proportion of patients with influenza A virus infection than type B were observed. The influenza activity started in week 47/2019, and influenza positivity rate was ≥ 50% for 2 weeks (05–06/2020) rather than 5–8 weeks in the previous five seasons. In many countries a rapid reduction in sentinel reports and the highest influenza activity was observed in weeks 09–13/2020. Reporting was reduced from week 14/2020 across the Region coincident with the onset of widespread circulation of SARS-CoV-2.

**Conclusions:**

Overall, influenza type A viruses dominated; however, there were varying patterns across the Region, with dominance of B/Victoria-lineage viruses in a few countries. The COVID-19 pandemic contributed to an earlier end of the influenza season and reduced influenza virus circulation probably owing to restricted healthcare access and public health measures.

## Introduction

Influenza viruses cause seasonal epidemics with high burden and severity, as well as pandemics. Representing a threat to public health, influenza is one of the most thoroughly monitored diseases globally [1-8]. The World Health Organization Regional Office for Europe (WHO/Europe) and the European Centre for Disease Prevention and Control (ECDC) jointly coordinate influenza surveillance in the European Region and present these data each week on the FluNewsEurope website [9,10]. The surveillance objectives are: to collect data to characterise the circulating influenza viruses, to determine the timing of influenza activity, to describe the most affected age and risk groups and the overall severity of disease, and to evaluate interventions (in particular vaccination) on a subnational, national, WHO European Region and global level [11,12].

Countries, territories and areas (hereafter referred to as countries) report syndromic, clinical and virological influenza surveillance data. These include data from sentinel sources, where a representative number of primary care physicians systematically collect respiratory specimens for laboratory PCR testing from a subset of patients with either influenza-like illness (ILI) or acute respiratory infection (ARI) [13-17]. Some countries such as Norway use consultation data from a database/register which covers the majority of the population. Following the 2009 influenza pandemic, the implementation of hospital-based surveillance was encouraged to inform national severity assessments [18].

The introduction of the new coronavirus disease (COVID-19) into Europe in late January 2020 and the response measures to that pandemic during the northern hemisphere influenza season had unprecedented effects on both influenza epidemiology and surveillance [19,20]. The aim of this manuscript is to describe the epidemiology, virology and severity of the 2019/20 influenza season and to assess the impact of the COVID-19 pandemic on the influenza season and the routine monitoring systems for influenza in the WHO European Region. We aim to understand and assess the changes that impact influenza surveillance in order to inform public health for future actions.

## Methods

### Data collection

Countries report weekly epidemiological and virological influenza data to The European Surveillance System (TESSy) hosted at ECDC. The time period for the influenza season is between week 40 and week 20 of the following year. The data snapshot was taken on 15 October 2020, and five previous seasons are included in the analysis for comparison.

### Primary care ILI or ARI surveillance

From a subset of patients with ILI or ARI visiting sentinel outpatient facilities, specimens from the upper respiratory tract were collected and tested for influenza viruses at national influenza centres or reference laboratories. The consultation rate for ILI or ARI was calculated as the number of patients per 100,000 population. The number of specimens that tested positive in PCR assays (with type and subtype determination) for influenza virus among the total number of specimens tested was used to calculate the percentage positive. A percentage of 10% positivity or higher was used as a threshold to determine the start of seasonal influenza activity and a percentage of 50% or higher as indicator for high influenza activity. Weekly proportions were calculated for each influenza virus type and subtype, using as the denominator either the total number of influenza-positive specimens or those testing positive for type A or type B viruses. The vast majority of influenza data on type A viruses only include haemagglutinin (HA) typing and no information about the neuraminidase (NA) type. We therefore only refer to influenza A(H1)pdm09 and A(H3) viruses in this analysis.

### Hospital-based surveillance 

In the European Region, some countries report case-based data from hospitalised laboratory-confirmed influenza cases (intensive care units (ICU) or wards other than ICU (non-ICU)), while others, mostly in the south-eastern part of the Region, perform hospital-based sentinel surveillance of severe acute respiratory infections (SARI) following a predefined syndromic clinical case definition [21]. A subset of all SARI cases is tested for influenza and other respiratory viruses.

Eighteen countries in the south-eastern and eastern part of the Region reported SARI-based hospital data (these countries are listed in the Supplement) [22]. Fourteen countries reported hospital data on laboratory-confirmed influenza cases from ICU and seven countries from non-ICU wards (countries listed in the Supplement). We used these data to describe disease severity by number of hospitalisations and fatalities according to virus type/subtype and age group.

### Ethical statement

Ethical approval was not required for this study. Data collection is conducted as part of the routine surveillance of communicable diseases with each country being responsible for the data collection according to national law.

## Results

### Primary care-based surveillance

**Figure 1:**
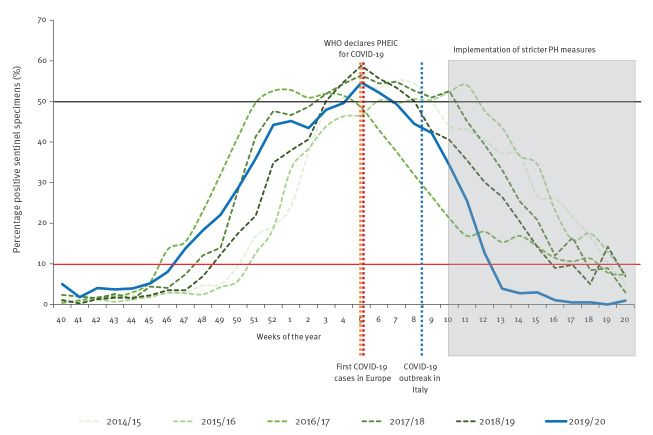
Percentage of sentinel specimens from patients with influenza-like illness or acute respiratory infection testing positive for influenza viruses per season, by week, European Region, 2014/15–2019/20

COVID-19: coronavirus disease; PH: public health response; PHEIC: public health event of international concern.

The 2019/20 influenza epidemic started in week 47/2019 (Figure 1), when sentinel detections crossed the 10% positivity threshold and peaked in week 05/2020 (55%). Positivity remained ≥ 10% until week 12, for 18 consecutive weeks, with a sharp decline between weeks 10–13. Seasonal activity was 1 week shorter than in the previous five seasons (range for seasons 2014/15 to 2018/19: 19–25 weeks). Positivity levels above 50% were reached for just two consecutive weeks (05/2020 and 06/2020), while the period of high influenza activity lasted 5–8 weeks in the previous five seasons (Figure 1).

10% line: 10% or higher was used as a threshold to determine the start of seasonal influenza activity; 50% line: 50% or higher was used as indicator for high influenza activity. Measures taken from [31].

**Figure 2:**
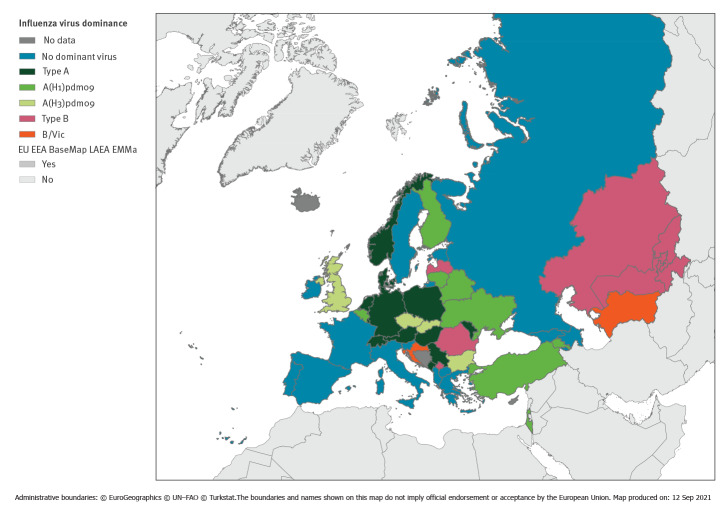
Dominant influenza virus types/subtypes, European Region, 2019/20 (n = 17,905)**

Influenza type A virus accounted for 64% (n = 11,471) and influenza B for 36% (n = 6,434) of the 17,905 detections in sentinel specimens from primary care. The overall number of influenza detections was similar to previous seasons (2014/15: 16,076; 2015/16: 19,512; 2016/17: 16,445; 2017/18: 22,321; 2018/19: 17,000). During 2019/20, influenza type A virus subtypes, A(H1)pdm09 (34.6%; n = 6,195) and A(H3) (23.9%; n = 4,279), and type B virus (35.9%; 6,434) co-circulated in the Region; 5.6% of the type A viruses were reported without subtype. Among the 39.4% type B viruses with lineage determination (2,532/6,434), the vast majority (99.1%; 2,509) were reported as B/Victoria. Co-circulation of influenza A(H1)pdm09 and A(H3) viruses was observed in the previous seasons 2018/19, and viruses of the B/Victoria lineage circulated previously in 2015/16 [23]. The pattern of virus dominance varied across countries with few reporting influenza type B dominance (Figure 2).

Dominant virus: proportion ≥ 60% of viruses reported for this virus type, subtype or lineage of all viruses reported.

### Hospital-based surveillance

#### Severe acute respiratory infections

Overall, 38,914 SARI cases were reported from 18 countries in the 2019/20 season. Of 9,751 patients swabbed and tested for influenza, 2,917 (29.9%) were positive. Slightly more influenza type A viruses were detected than type B (1,633 vs 1,284; 56.0% vs 44.0%), and of the 1,376 subtyped type A viruses, 61.2% (n = 842) were influenza A(H1)pdm09 and 38.8% (n = 534) A(H3).

#### Laboratory-confirmed influenza in non-ICU wards

**Figure 3:**
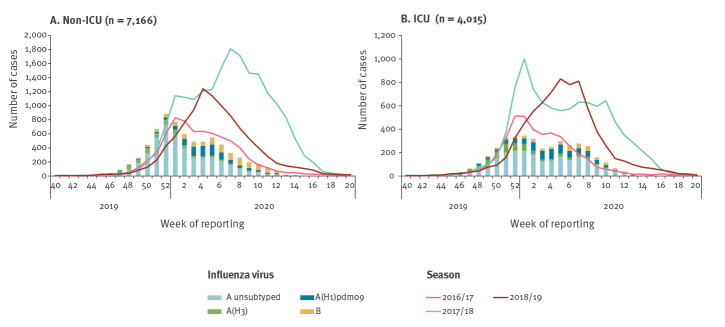
Number of hospitalised laboratory-confirmed influenza cases on non-ICU and ICU wards by week of reporting and type/subtype, 2019/2020 compared with total number in seasons 2016/17–2018/19 (n = 11,181)

ICU: intensive care unit; non-ICU: other non-ICU wards.

In 2019/20, 7,235 laboratory-confirmed cases of influenza on non-ICU wards were reported by seven countries (90.6%; n = 6,552 of the cases were reported by Ireland and Spain). This was less than in the seasons 2017/18 and 2018/19 with 19,480 and 10,148 cases, respectively (Figure 3). However, it was comparable to the 7,154 cases in the season 2016/17. Of the 7,233 cases with known age and virus type or subtype/lineage, 15.6% (n = 1,129) were 0–4 years-old, 11.5% (n = 835) 5–14 years-old, 30.9% (n = 2,234) 15–64 years-old and 42.0% (n = 3,035) were 65 years or older. Most patients were diagnosed with influenza virus type A infection and 60.5% (n = 4,373) of them were reported as not subtyped. Of the 1,708 subtyped viruses, 58.7% (n = 1,002) were influenza A(H1)pdm09 and 41.3% (n = 706) A(H3).

Five countries reported 334 deaths, and 88.3% (n = 295) of the deaths were in patients 65 years and older. Among the fatal cases, 91.6% (n = 306) were due to influenza A virus infection and 74.6% (97/130) of the subtyped influenza A viruses were A(H1)pdm09. Twenty of the 28 reported fatalities due to type B virus infection were 65 years and above.

#### Laboratory-confirmed influenza in ICU

In season 2019/20, 4,015 laboratory-confirmed cases, including 338 fatalities, were reported from ICU in 11 countries. Most cases in ICU (85.8%; 3,444/4,015) were reported by France, Spain and England. Similar to non-ICU cases, the numbers were comparable to season 2016/17 (n = 4,114) but lower than in 2017/18 (n = 9,725) and 2018/19 (n = 7,424; Figure 3). Among all cases, 90.0% (n = 3,615) were due to influenza type A infection, of which 64.8% (n = 2,344) remained unsubtyped. Of the 1,271 subtyped viruses, 58.5% (n = 744) were influenza A(H1)pdm09 and 41.5% (n = 527) were influenza A(H3). Of 2,200 patients with known age and type or subtype/lineage, 48.7% (n = 1,072) were 15–64 years-old and 36.8% (n = 810) were 65 years and older.

Of the 338 cases that died in ICU, 52.1% (n = 176) were 65 years and older. Influenza type A virus infection was reported in 92.6% (n = 313); 96 (28.4%) were due to influenza A(H1)pdm09, 37 (10.9%) to influenza A(H3) and 180 (53.3%) were reported without a virus subtype. Influenza type B virus was detected in 25 patients who died, 12 of whom were 15–64 years-old, nine were aged 65 or older and four were younger than 5 years.

### Impact of COVID-19 measures on syndromic primary care sentinel and hospital-based surveillance

An analysis of data completeness showed that an increasing number of countries did not report influenza data from week 12/2020 or 13/2020 onwards (Supplementary Figures S1- S6). Three countries stopped reporting consultation data before week 10/2020 (England, Iceland and Kyrgyzstan); seven stopped reporting between weeks 10/2020 and 15/2020 (Albania, Austria, France, Switzerland, Uzbekistan, Turkmenistan and Kosovo*). Following the season peak, several countries had noticeably lower ARI consultation rates at the end of the season compared with previous years. Similarly, several countries showed a late season increase in ILI rates with a shift in the affected age groups (i.e. Belgium, France, Ireland, Lithuania, Luxembourg and Norway; Supplementary Figures S7–S8).

Sentinel virological data reporting was stopped by nine countries before week 10/2020 (Croatia, Czechia, England, Greece, Israel, Malta, North Macedonia, Serbia and Turkey; Supplementary Figure S9). An additional 13 stopped reporting between weeks 10/2020 and 15/2020: Albania, Austria, Belarus, Bulgaria, France, Hungary, Kazakhstan, Kyrgyzstan, Montenegro, Switzerland, Uzbekistan, Turkmenistan and Kosovo*. Other countries such as Ireland continued reporting but did not have a positive sentinel detection after week 13 because the sentinel GP virological surveillance was disrupted because of COVID-19 and was reinstated only in November 2020.

**Figure 4:**
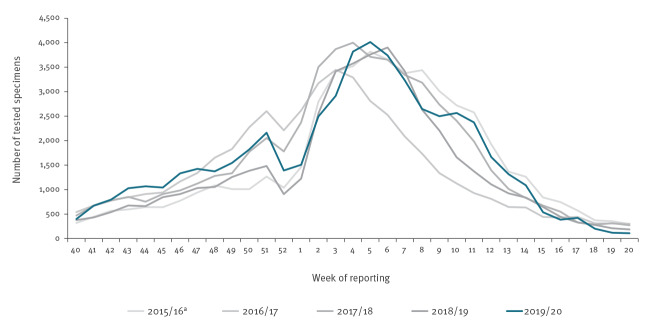
Total specimens from influenza sentinel primary care surveillance tested for influenza viruses by week and season, European Region, seasons 2015/16–2019/20 (n = 53,684)

^a^ Week 53 in 2015/16 excluded.

The overall pooled number of tested specimens for the season was comparable to the five previous seasons (2014/15: 43,473; 2015/16: 52,682; 2016/17: 47,148; 2017/18: 55,171; 2018/19: 46,234 and 2019/20: 53,828). However, a more detailed look at the data by country showed that some stopped sentinel testing for influenza between weeks 6/2020 and 12/2020, while others such as the Netherlands maintained their testing capacities at comparable levels. In addition, Denmark, and to a lesser extent Sweden, increased their testing of sentinel specimens compared with previous seasons (Figure 4 and Supplementary Figure S10). Denmark contributed 0–2% of the pooled number of specimens between weeks 40/2019 and 12/2020, but 20–52% of the total tested specimens from week 13/2020 onwards. Likewise, Sweden, which typically reported 1–3% of the total samples, increased the number of tested sentinel specimens between weeks 11/2020 and 15/2020 and contributed 10–25% of the total samples tested across the Region.

One country stopped SARI reporting before week 10/2020 (Kyrgyzstan) and an additional six stopped reporting between weeks 10/2020 and 15/2020 (Albania, Belarus, Kosovo*, Montenegro, North Macedonia and Turkmenistan).

## Discussion

In the WHO European Region, the 2019/20 influenza season started in late November 2019 and peaked in the first week of February. Mixed circulation of influenza A(H1)pdm09, A(H3) and B/Victoria virus, with different patterns of dominance, was observed across the countries. Influenza A(H3) viruses are known to disproportionately affect persons aged 65 years and older, while patients infected with influenza A(H1)pdm09 viruses tend to be slightly younger and B/Victoria viruses are more common in children [24-27]. Most fatal outcomes in hospitalised patients occurred in elderly people, which was also observed in German [28] and French national data [29]. The co-circulation of both influenza A virus subtypes could have contributed to the all-cause excess mortality observed between weeks 01/2020 and 03/2020 in the age group 65 years and older [30].

In late February, when influenza activity had passed the peak and begun to decline slowly in the WHO European Region, the number of COVID-19 cases increased sharply. The first strict public health measures (e.g. border closures, lockdowns) were implemented around week 11/2020 [31]. Movement restrictions and stay-at-home orders decreased access to routine primary healthcare and sentinel sites and disrupted diagnostic capacities. In addition, response efforts led to reorganisation of healthcare access and testing resources, resulting in decreased syndromic consultation data and influenza detections. With the onset of community transmission of COVID-19 during March 2020, changes in healthcare-seeking behaviour, reducing the swabbing of patients presenting with ARI, as well as challenges with specimen shipment led to reductions in influenza-related indicators from this period onwards. We also need to highlight that many countries maintained or increased their influenza surveillance activities throughout the COVID-19 pandemic (until the present day). Data that were reported indicated that influenza activity had fallen substantially. Reported influenza cases became increasingly sparse over the following months. The introduction of a novel respiratory pathogen, severe acute respiratory syndrome coronavirus 2 (SARS-CoV-2), resulting in the widespread use of social and physical distancing measures throughout the Region probably contributed to a sharp reduction in influenza virus circulation and an abrupt end to the influenza season. Despite the limited data from sentinel systems, additional data on influenza circulation from non-sentinel sources not displayed in this paper, showed the same decrease in influenza detections despite continued testing throughout the spring and summer, confirming an overall lower influenza circulation following the introduction of COVID-19-related measures [32]. An analysis of the impact of pandemic public health measures on influenza circulation in the Scandinavian countries describes the rapid end of the influenza epidemic in these countries and the measures implemented [33]. Similar effects on influenza surveillance and epidemiology were observed globally, and influenza detections continued to be at a low level at least until September 2021 [34,35]. In addition, the usual seasonal influenza epidemic in the southern hemisphere was strongly impacted in 2020, with just a few influenza detections overall [36,37].

In the European Region, many countries have modified their sentinel and hospital influenza surveillance systems to also detect SARS-CoV-2, and the concept of clinical ARI surveillance, which is a broader case definition than ILI to better cover other respiratory viruses, may have helped to integrate SARS-CoV-2 in the virological sentinel surveillance. The ECDC and WHO/Europe have published interim guidance for the European Region on how to include SARS-CoV-2 testing during the 2020/21 season and suggested to keep the ARI, ILI and SARI case definitions as in previous seasons [38]. Despite continued high levels of testing, influenza circulation remained low after week 20/2020 and during the 2020/21 season in the European Region [32,39]. The ability and value of sentinel surveillance to include SARS-CoV-2 monitoring in addition to influenza needs to be evaluated. There is much uncertainty about the timing and level of influenza circulation as well as the virus subtype or lineage (or even clade) in future seasons, which is also dependent on the global SARS-CoV-2 situation, related measures and influenza vaccine coverage in the population. Guidance for the upcoming season 2021/22 may need to consider different aspects including historical comparability of data, while being flexible enough to adjust to the situation and capacities in the countries.

Vaccination remains one of the best interventions to prevent influenza, and there are renewed efforts to increase vaccine uptake for eligible groups. Vaccine coverage data across countries are not available for the latest seasons; however, coverage rates across the countries have not reached the 75% target in the European Union for the older age groups and are generally lower in the general population until 2016/17; France for instance reported 47.8% coverage among people at risk for the season 2020/21 [29,40-42]. Even with similar or slightly higher vaccination rates, it is very unlikely that vaccination is the factor associated with the observations in the 2019/20 season. Maintaining influenza surveillance with the collection and sharing of detailed virus characterisation is instrumental in order to support on the global level the selection of the seasonal influenza vaccine strains for the coming years.

## Conclusions

The 2019/20 influenza season was characterised by co-circulation of influenza A(H1)pdm09, A(H3) and B/Victoria viruses, with different pattern of dominance in the countries across the WHO European Region. The COVID-19 pandemic impacted influenza epidemiology and surveillance, in that the 2019/20 influenza epidemic ended earlier than the five previous seasons. 

## *Note

This designation is without prejudice to positions on status, and is in line with UNSCR 1244/99 and the IСJ Opinion on the Kosovo declaration of independence.

## **Erratum

The legend of Figure 2 has been corrected to have light green labelled ‘A(H3)’ rather than ‘A(H3)pdm09’ as originally published. This change was made on 8 October 2021.

## Supplementary Data

890000 Supplement
