# Erratum for Euro Surveill. 2021;26(40)

**DOI:** 10.2807/1560-7917.ES.2021.26.41.211014e

**Published:** 2021-10-14

**Authors:** Eurosurveillance editorial team

**Affiliations:** 1 European Centre for Disease Prevention and Control (ECDC), Stockholm, Sweden

The legend of Figure 2 in the article ‘Spotlight influenza: The 2019/20 influenza season and the impact of COVID-19 on influenza surveillance in the WHO European Region’ by Adlhoch et al., published on 7 October 2021 has been corrected to have light green labelled ‘A(H3)’ rather than ‘A(H3)pdm09’ as originally published. This change was made on 8 October 2021. 

