# Supplementary material for: Spotlight influenza: The 2019/20 influenza season and the impact of COVID-19 on influenza surveillance in the WHO European Region
Source: Euro Surveill. 2021 Oct 7;26(40):2100077. doi: 10.2807/1560-7917.ES.2021.26.40.2100077 (PMC8511754; doi:10.2807/1560-7917.ES.2021.26.40.2100077)

Online supplement information:

This supplementary material is hosted by *Eurosurveillance* as supporting information alongside the article Spotlight influenza: The 2019/20 influenza season and the impact of COVID-19 on influenza surveillance in the WHO European Region, on behalf of the authors, who remain responsible for the accuracy and appropriateness of the content. The same standards for ethics, copyright, attributions and permissions as for the article apply. Supplements are not edited by *Eurosurveillance* and the journal is not responsible for the maintenance of any links or email addresses provided therein.

Following countries, territories and areas reported SARI-based hospital data in 2019/20: Albania, Armenia, Azerbaijan, Belarus, Bosnia and Herzegovina, Georgia, Kazakhstan, Kyrgyzstan, Montenegro, North Macedonia, Republic of Moldova, Russian Federation, Serbia, Tajikistan, Turkmenistan, Ukraine, Uzbekistan, and Kosovo[1].

Fourteen countries, territories and areas reported hospital data on laboratory-confirmed influenza cases from ICUs: Czechia, Finland, France, Ireland, Romania, Russian Federation, Slovakia, Spain, Sweden, Ukraine, and the United Kingdom.

Seven countries, territories and areas from non-ICU wards: Czechia, Ireland, Romania, Russian Federation, Slovakia, Spain, and Ukraine.

[1] All references to Kosovo should be understood to be in the context of the United Nations Security Council resolution 1244 (1999).

Supplementary online figures:

Figure S1. INFLICLIN reporting by week – number of non-reporting countries (N=54 countries, territories and regions) by ISO week between 40/2019 and 20/2020, WHO European Region

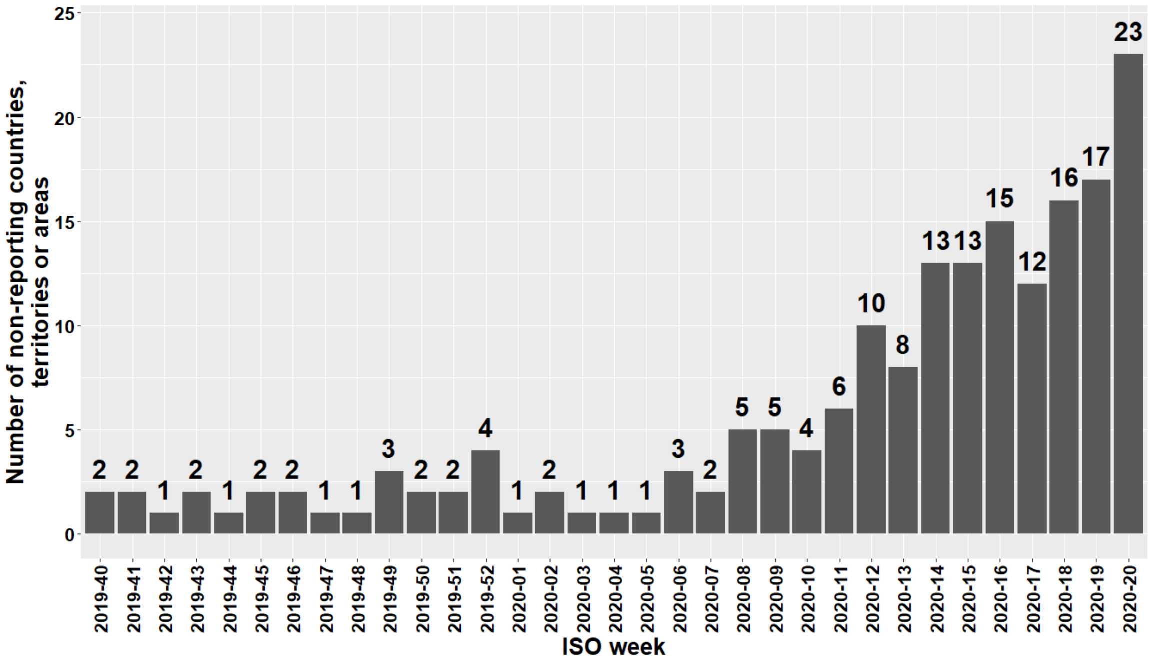

Figure S2. INFLIVIR reporting by week – number of non-reporting countries (N=54 countries, territories and regions) by ISO week between 40/2019 and 20/2020, WHO European Region

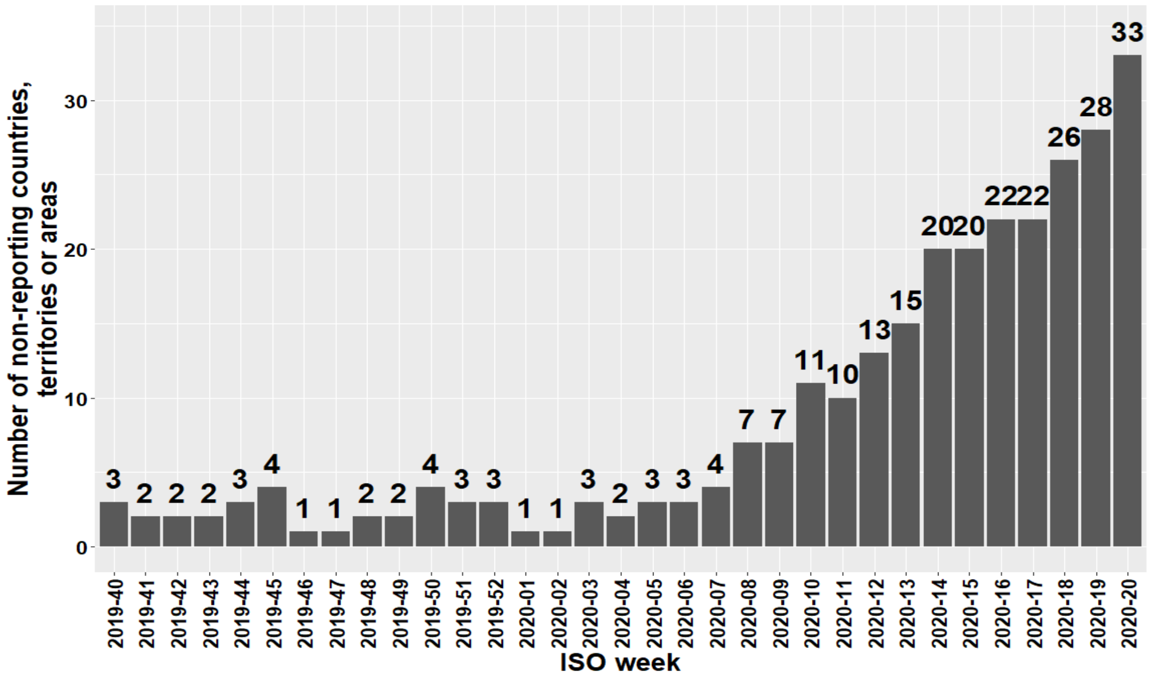

Figure S3. INFLSARIAGGR reporting by week – number of non-reporting countries (N=54 countries, territories and regions) by ISO week between 40/2019 and 20/2020, WHO European Region

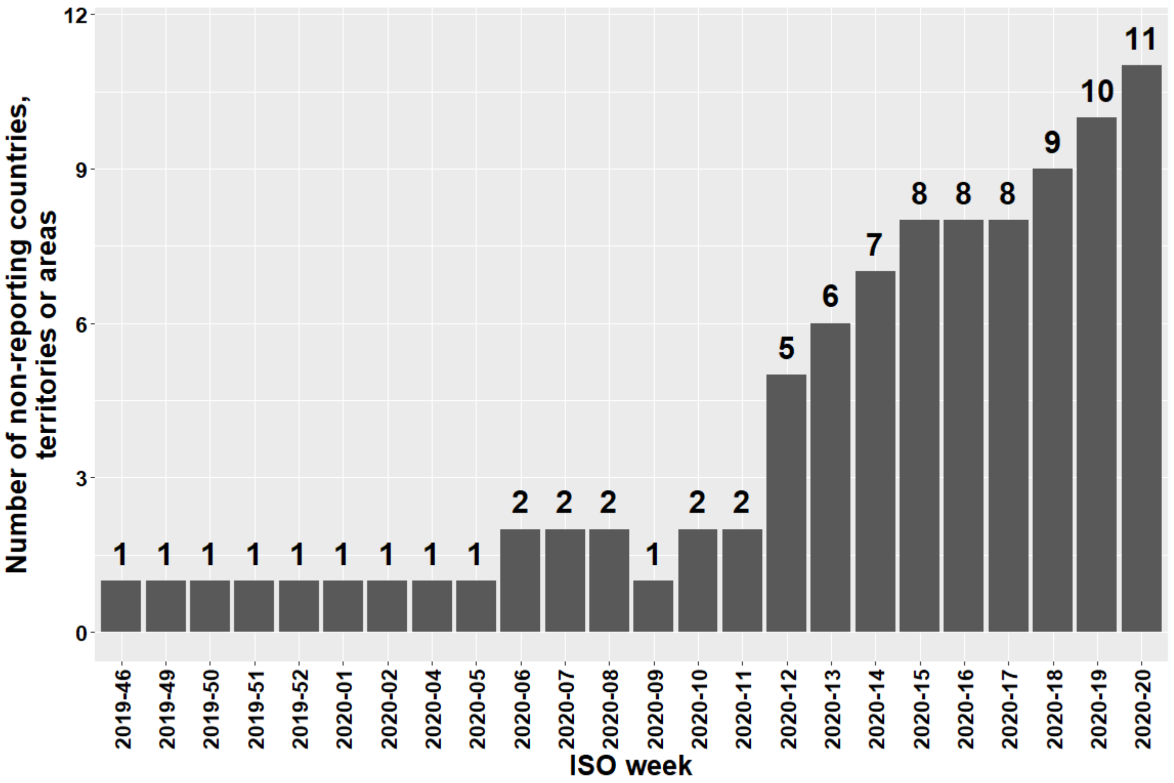

Figure S4. INFCLIN reporting status of countries by ISO week between 40/2019 and 20/2020, WHO European Region

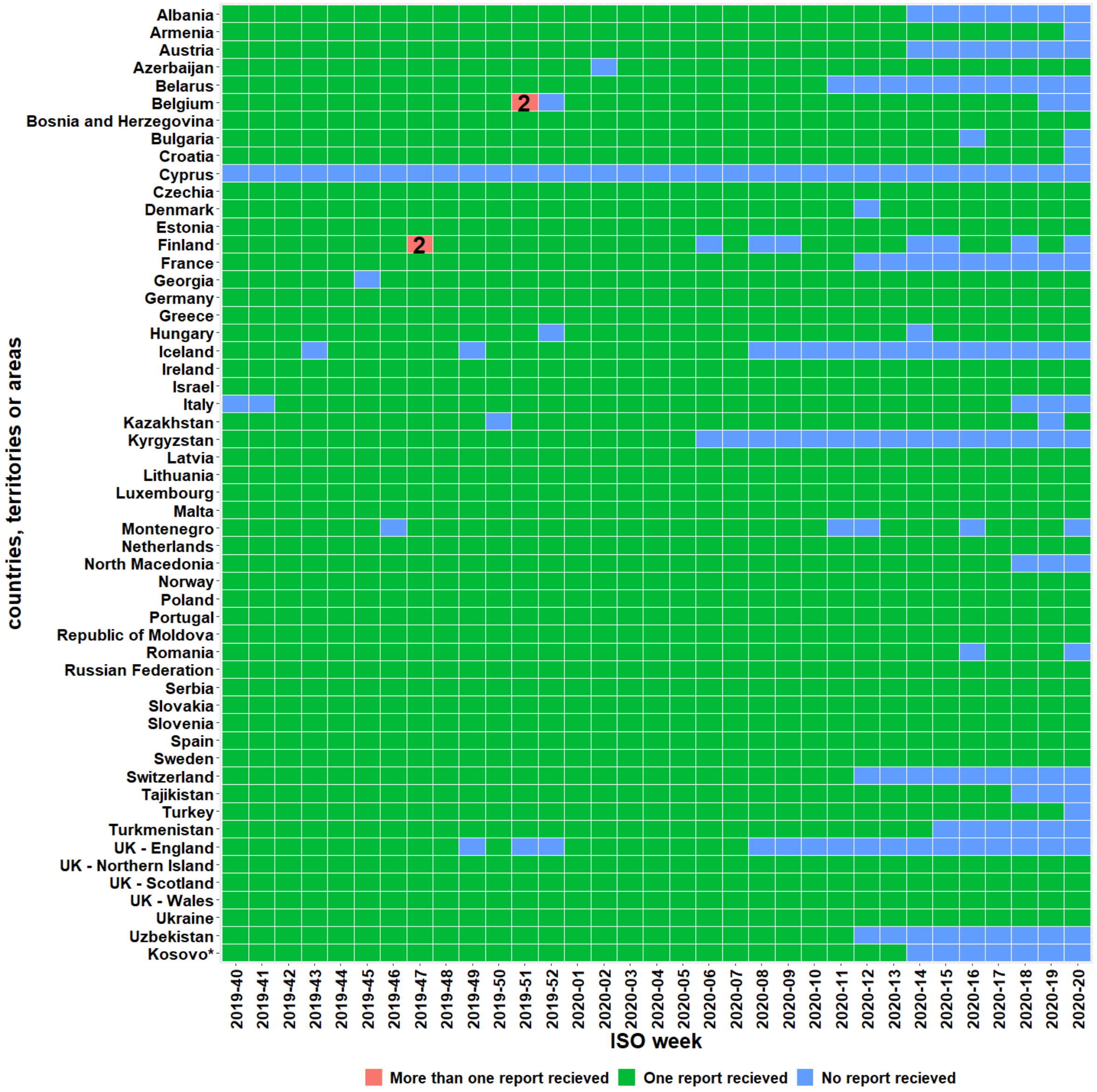

Figure S5. INFLIVIR reporting status of countries by ISO week between 40/2019 and 20/2020, WHO European Region

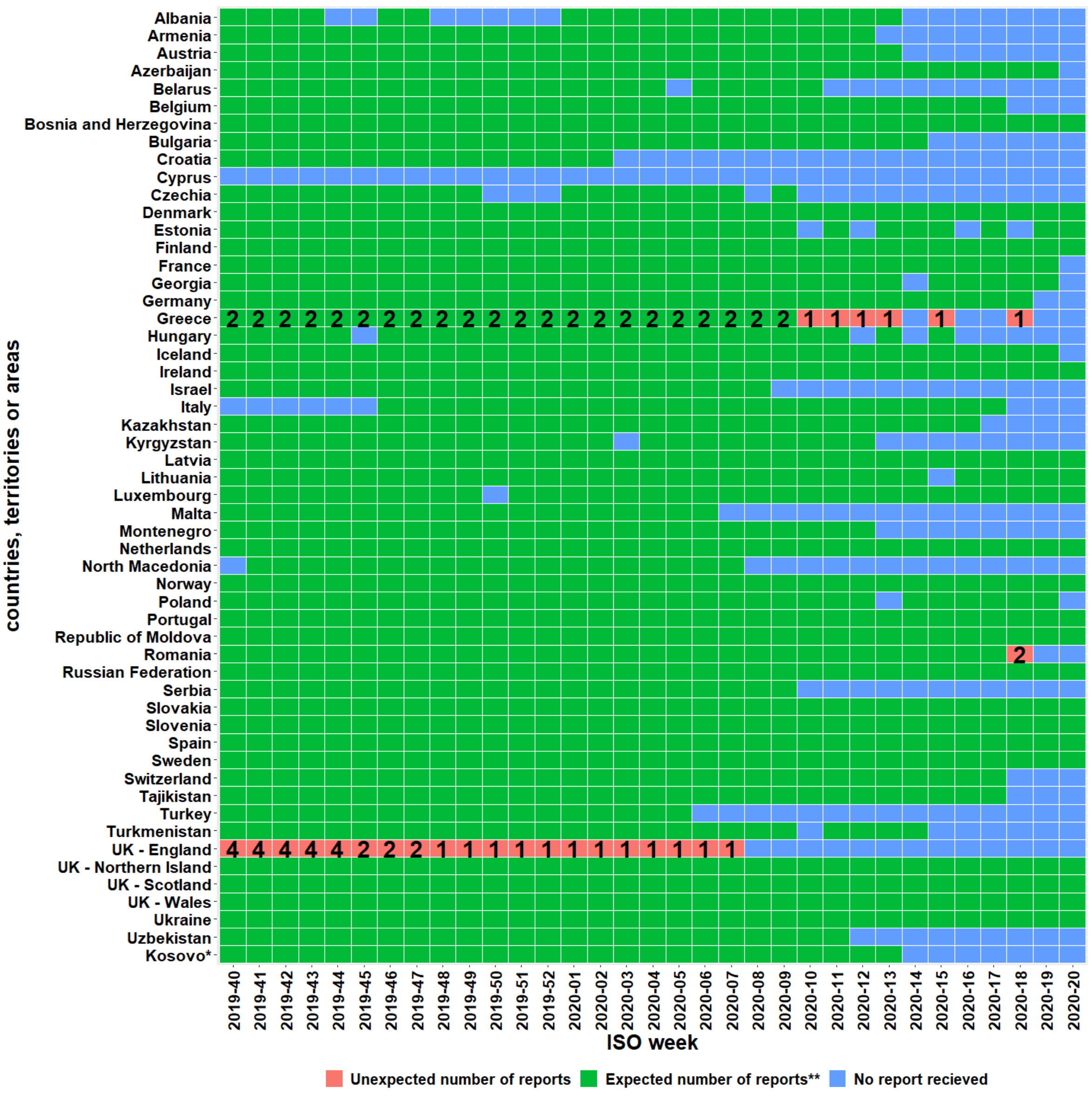

Figure S6. INFLSARIAGGR reporting status of countries by ISO week between 40/2019 and 20/2020, WHO European Region

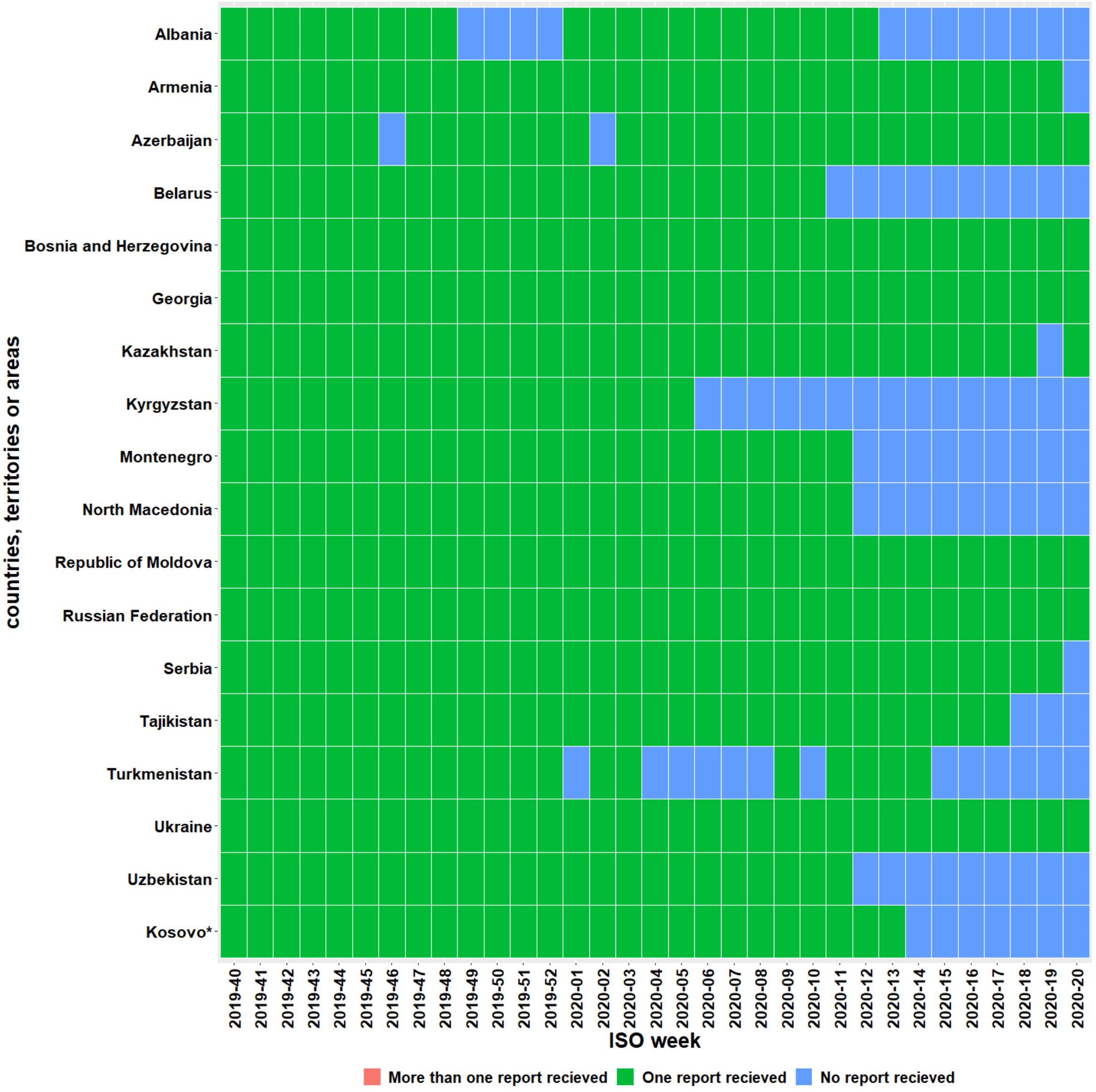

Figure S7. Consultation rate for influenza-like illness (ILI) per 100,000 population by week of reporting, age-group (A) and season (B), Belgium 2019/20 (two peaks in ILI)

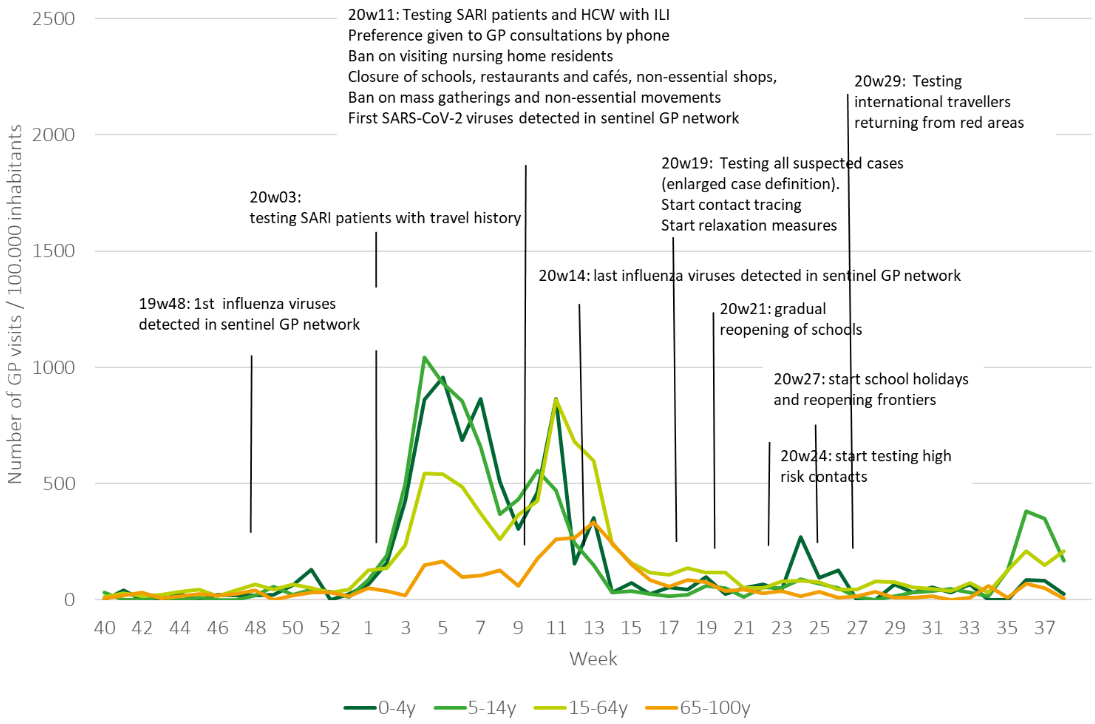

A

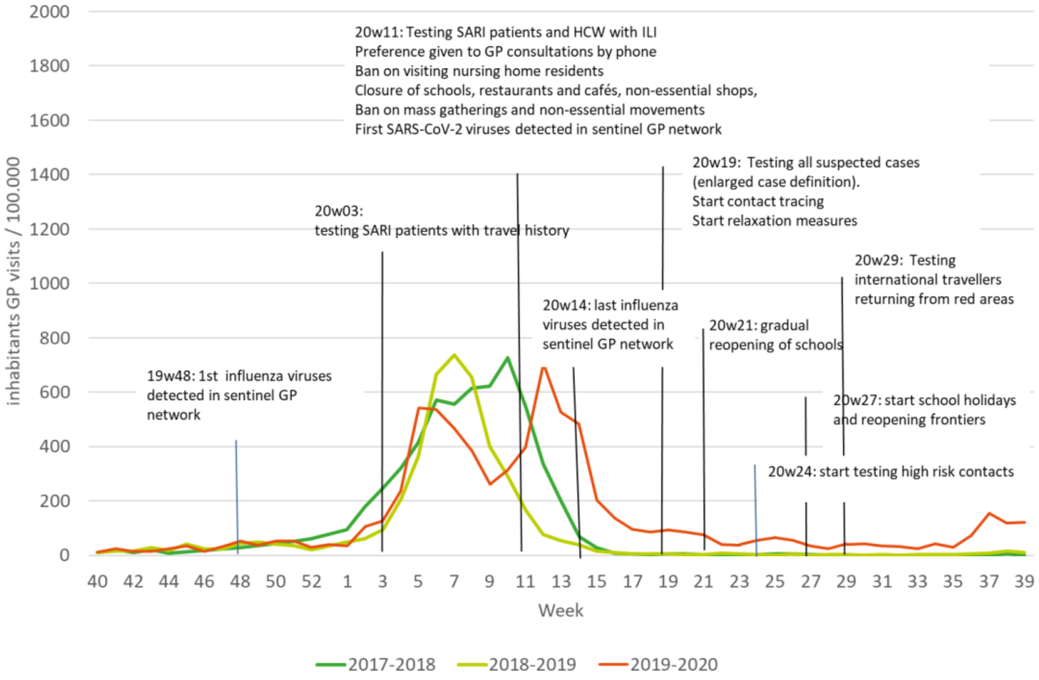

B

Figure S8. Consultation rate for influenza-like illness (ILI) per 100,000 population by week of reporting, season and age-group, Ireland 2019/20 (second peak*)

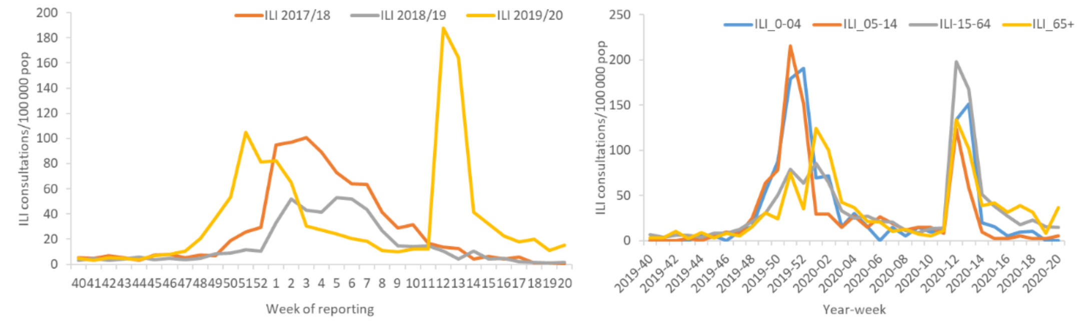

* The second ILI peak for Ireland was more a reflection of COVID activity as very little influenza testing undertaken at that time

Figure S9. Sentinel tests Turkey by week of reporting and season– example of abrupt cessation of sentinel testing

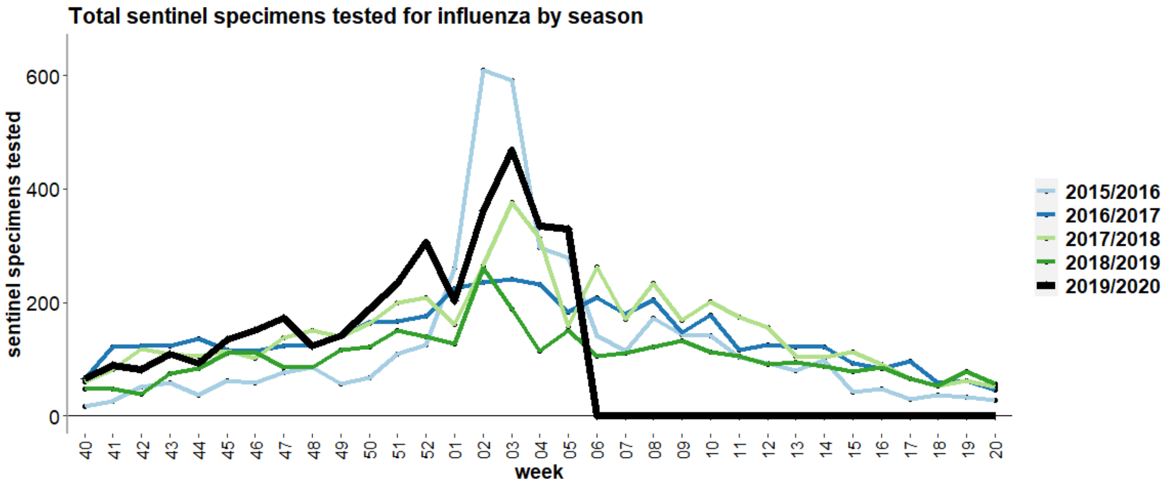

Figure S10. Total sentinel specimens tested Denmark by week of reporting and season – example of increased testing

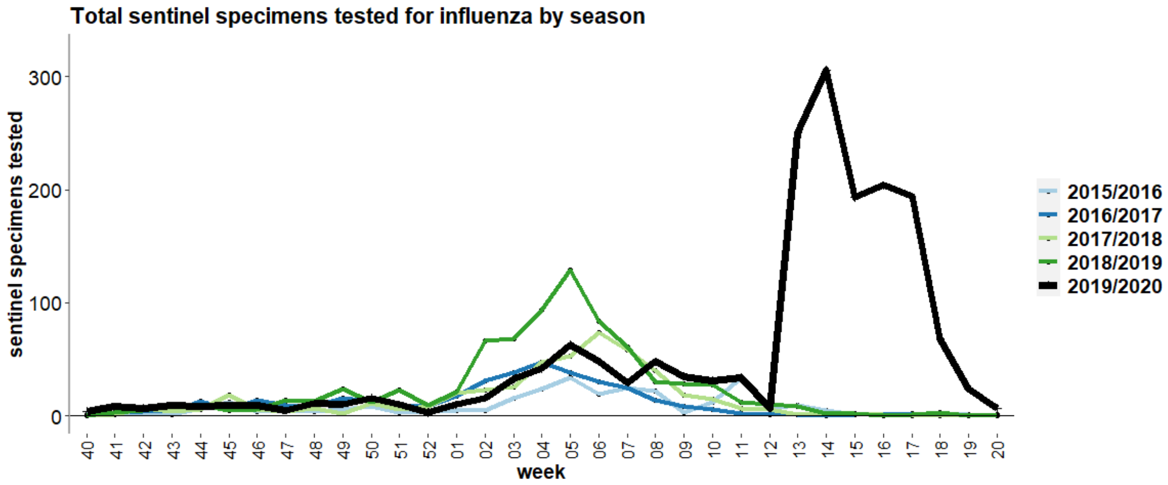

Supplement: Supplement [file 21-00077_ADLHOCH_supplement.pdf]
